# Sex Differences in the Association Between Measures of General and Central Adiposity and the Risk of Myocardial Infarction: Results From the UK Biobank

**DOI:** 10.1161/JAHA.117.008507

**Published:** 2018-02-28

**Authors:** Sanne A. E. Peters, Sophie H. Bots, Mark Woodward

**Affiliations:** ^1^ The George Institute for Global Health University of Oxford Oxford United Kingdom; ^2^ The George Institute for Global Health University of New South Wales Sydney Australia; ^3^ Department of Epidemiology Johns Hopkins University Baltimore MD

**Keywords:** adipose tissue, men, myocardial infarction, women, Obesity, Risk Factors, Women, Epidemiology, Cardiovascular Disease

## Abstract

**Background:**

There are substantial differences in the distribution of adipose tissue between women and men. We assessed the sex‐specific relationships and their differences between measures of general and central adiposity and the risk of incident myocardial infarction (MI).

**Methods and Results:**

Between 2006 and 2010, the UK Biobank recruited over 500 000 participants aged 40 to 69 years across the United Kingdom. During 7 years of follow‐up, 5710 cases of MI (28% women) were recorded among 265 988 women and 213 622 men without a history of cardiovascular disease at baseline. Cox regression models yielded adjusted hazard ratios for MI associated with body mass index, waist circumference, waist‐to‐hip ratio, and waist‐to‐height ratio. There was an approximate log‐linear relationship between measures of general and central adiposity and the risk of MI in both sexes. A 1‐SD higher in body mass index, waist circumference, waist‐to‐hip ratio, and waist‐to‐height ratio, respectively, were associated with hazard ratios (confidence intervals) for MI of 1.22 (1.17; 1.28), 1.35 (1.28; 1.42), 1.49 (1.39; 1.59), and 1.34 (1.27; 1.40) in women and of 1.28 (1.23; 1.32), 1.28 (1.23; 1.33), 1.36 (1.30; 1.43), and 1.33 (1.28; 1.38) in men. The corresponding women‐to‐men ratios of hazard ratios were 0.96 (0.91; 1.02), 1.07 (1.00; 1.14), 1.15 (1.06; 1.24), and 1.03 (0.97; 1.09).

**Conclusions:**

Although general and central adiposity measures each have profound deleterious effects on the risk of MI in both sexes, a higher waist circumference and waist‐to‐hip ratio conferred a greater excess risk of MI in women than in men. Waist‐to‐hip ratio was more strongly associated with the risk of MI than body mass index in both sexes, especially in women.

**Clinical Perspective**

**What Is New?**

- Measures of central adiposity, but not of general adiposity, were more strongly associated with the risk of myocardial infarction in women than in men.
- Measures of central adiposity, particularly waist‐to‐hip ratio, were more strongly associated with the risk of myocardial infarction than general adiposity, especially among women.
- Thus, this study suggests that the sex dimorphism in the quantity and distribution of adipose tissue not only results in differences in body shape between women and men but may also have differential implications for the risk of myocardial infarction in later life.

**What Are the Clinical Implications?**

- Compared with body mass index, measures of central adiposity may be a better indicator of the risk of myocardial infarction associated with adiposity in women and also in men.
- Insight into the sexual dimorphism in adiposity will yield insights into the biological mechanisms and could inform sex‐specific interventions to treat and halt the obesity epidemic.

## Introduction

Excess adipose tissue is a major, and increasingly common, risk factor for chronic diseases, including myocardial infarction (MI), the leading cause of death worldwide.^1^, ^2^ In 2016, the World Health Organization estimated that more than 40% of women and 39% of men worldwide were overweight (defined as a body mass index [BMI] of >25 kg/m^2^) and that over 15% of women and 11% of men were obese (BMI >30 kg/m^2^).^3^

BMI is the most widely used measure to assess the prevalence of overweight and obesity across populations and to quantify the detrimental effects of excess adipose tissue on the risk of MI in later life. However, BMI is a measure of general adiposity and does not discriminate between adipose tissue present in visceral and subcutaneous areas. Yet, as compared with subcutaneous adipose tissue, visceral adipose tissue is more metabolically active, is closely related to insulin resistance, and may be more strongly associated with cardiometabolic risk.^4^, ^5^ Measures of central adiposity and body composition, including waist circumference, waist‐to‐hip ratio, and waist‐to‐height ratio, may, therefore, be better suited than BMI for quantifying the etiological relationship between adiposity and cardiovascular disease. Although some studies have reported that measures of central adiposity are more strongly related to cardiovascular risk than BMI,^6^, ^7^, ^8^, ^9^, ^10^ others have found minimal differences across anthropometric measures and reported that each measure was similarly associated with the risk of future MI.^11^, ^12^

There are substantial sex differences in body fat distribution, with a predominance of subcutaneous fat in women and visceral fat in men.^13^, ^14^ Although the association between BMI and the risk of MI is broadly similar between women and men,^15^ it is conceivable that there is a sex differential in the effects of central adiposity on the risk of MI—with potentially stronger effects in women than men. However, most previous studies did not report the associations between measures of general and central adiposity and the risk of MI separately for women and men. Even when sex‐specific results on multiple anthropometric measures were available, direct comparisons between measures for women and men separately were lacking.

The aim of this study was, thus, to directly assess the sex‐specific relationship between measures of general and central adiposity and the risk of incident MI among women and men without a prior history of cardiovascular disease included in the UK Biobank.

## Methods

### Transparency

This research has been conducted using the UK Biobank Resource (Application Number: 2495). Researchers can apply to use the UK Biobank resource by making their own application. The analytic methods and study materials will be made available to other researchers for purposes of reproducing the results or replicating the procedure on request.

### Study Population

The UK Biobank is a large prospective, population‐based cohort study.^16^, ^17^ Between 2006 and 2010 UK Biobank investigators sent postal invitations to over 9 million individuals registered with the UK's National Health Service who were aged 40 to 69 years and lived within ≈25 miles (40 km) of at least one of 22 assessment centers located throughout England, Wales, and Scotland. Over 500 000 women and men (5.5% response rate) consented to participate in the study and visited an assessment center between 2006 and 2010. Although the cohort is not representative of the general population, it is well designed to reliably detect generalizable associations between a wide range of baseline characteristics and health outcomes due to the sufficiently large numbers of participants across the full distribution of exposures. Participants provided informed consent electronically and completed questionnaires on their lifestyle, environment, and medical history, had physical and functional measures performed, and had samples of blood, urine, and saliva collected. UK Biobank has obtained Research Tissue Bank approval from its governing Research Ethics Committee, as recommended by the National Research Ethics Service. No separate ethics approval was required. Participants with a history of cardiovascular disease at baseline (n=20 100) or BMI <15 or >60 kg/m^2^ were excluded (n=2918).

### Adiposity Measures

Trained staff used standardized procedures and regularly calibrated equipment to obtain the body size measurements. Waist circumference at the level of the umbilicus was measured using a Wessex nonstretchable sprung tape measure. Hip circumference was measured using the same tape measure. Standing height was measured with a Seca 202 height measure after participants had removed their shoes. Body weight was measured using the Tanita BC‐418 MA body composition analyzer after shoes and heavy outer clothing were removed. BMI was calculated by dividing weight (kilograms) over height (meters) squared; waist‐to‐hip ratio was calculated by dividing the waist circumference by the hip circumference; and waist‐to‐height ratio was calculated by dividing the waist circumference by standing height.

### Study Outcomes

The study end point was the incidence of fatal or nonfatal MI, as defined by the UK Biobank. Follow‐up started at inclusion in the UK Biobank study (the baseline) and ended on March 1, 2016 or on the first fatal or nonfatal MI for all participants. Outcome adjudication involved linkage with hospital admissions data in England, Scotland, and Wales and national death register data to identify the date of the first known MI after baseline. Incident MI was defined by codes I21, I22, I23, I24.1 or I25.2 in the 10th revision of the *International Classification of Diseases* (*ICD‐10*).

### Statistical Analyses

Baseline characteristics are presented as means (standard deviation) for continuous variables and as percentages for categorical variables. Pearson correlation coefficients were obtained for each pair of adiposity measures. Repeat body size measures, which were collected on average 4 years after the baseline assessment in a reasonably representative sample of 20 277 individuals, were used to obtain the sex‐specific mean differences (standard deviation) in body size measures between the baseline assessment and first repeat assessment. Cox regression models were used to obtain hazard ratios (HRs) and 95% confidence intervals using MI associated with each of the adiposity measures, separately for women and men. Models were adjusted for age (continuous), Townsend deprivation index (continuous), and smoking status (categorical). The first 2 years of follow‐up were excluded from the analyses to minimize the effect of latent disease. HRs were derived in fifths of the distribution of the adiposity measure, apart from BMI, where predefined categories were used. In each case the reference group was chosen to be women in the second lowest adiposity measure group. Confidence intervals were estimated using floating absolute risks for comparisons involving more than 2 groups.^18^ Given that the relationships were approximately log‐linear, further analyses were conducted as the sexes‐combined HR per 1‐SD higher adiposity (for each measure). The sexes‐combined SDs were 4.76 for BMI, 13.4 for waist circumference, 0.09 for waist‐to‐hip ratio, and 0.08 for waist‐to‐height ratio. An interaction term was added to each model to evaluate whether the HRs differed between the sexes. Estimated differences in the ratios of HRs between measures of adiposity were computed, by sex, with 95% confidence intervals of the differences derived through bootstrapping with 500 replicates. Subgroup analyses were conducted to obtain the sex‐specific HRs and their ratios by age group (<60 years versus ≥60 years), socioeconomic status (Townsend deprivation index > −0.56 [“lower”] versus Townsend deprivation index ≤ −0.56 [“higher”]), and BMI (<25 kg/m² versus ≥25 kg/m^2^). Differences across subgroups were tested by adding, for the sex‐specific HRs, 2‐way interaction terms, and, for the women‐to‐men ratios, 3‐way interaction terms to the model. All analyses were performed using R version 3.3.1 (R Foundation, Vienna, Austria).

## Results

In total, 479 610 (55% women) individuals with a mean age of 56 years at recruitment were included (Table 1). The mean BMI was 27 kg/m^2^ in women and 28 kg/m^2^ in men. The mean waist circumference, waist‐to‐hip ratio, and waist‐to‐height ratio, respectively, were 85 cm, 0.82, and 0.52 in women and 97 cm, 0.93, and 0.55 in men. Correlation coefficients among BMI, waist circumference, and waist‐to‐height ratio were 0.88 and above for both sexes. Correlations with waist‐to‐hip ratio were 0.46 in women and 0.59 in men for BMI, 0.75 in women and 0.80 in men for waist circumference, and 0.75 in women and 0.80 in men for waist‐to‐height ratio (Table 2). Mean differences in body size measures between the baseline and repeat assessment were small and similar between the sexes (Table S1).

**Table 1:** Baseline Characteristics of Study Participants by Sex

	Women	Men
N	265 988	213 622
Age, y	56.3 (8.0)	56.4 (8.2)
Socioeconomic status, %		
Lower half	32.5	33.0
Higher half	67.5	67.0
Smoking status, %		
Never	59.9	50.1
Previous	31.3	37.8
Current	8.8	12.4
Body mass index, kg/m^2^	27.0 (5.2)	27.8 (4.2)
Waist circumference, cm	84.6 (12.5)	96.7 (11.2)
Hip circumference, cm	103.3 (10.3)	103.4 (7.5)
Standing height, cm	162.5 (6.3)	175.8 (6.8)
Weight, kg	71.4 (14.0)	85.8 (14.3)
Waist‐to‐hip ratio	0.82 (0.1)	0.93 (0.1)
Waist‐to‐height ratio	0.52 (0.1)	0.55 (0.1)

Values are means (standard deviation) for continuous variables and percentages for categorical variables.

**Table 2:** Pairwise Correlations between Body Mass Index, Waist Circumference, Waist‐to‐Hip Ratio, Waist‐to‐Height Ratio, Weight, Height, and Hip Circumference by Sex

	BMI	WC	WHR	WHtR	Weight	Height	Hip
BMI							
Women		0.876	0.457	0.885	0.917	−0.128	0.890
Men		0.876	0.592	0.884	0.882	−0.055	0.817
WC							
Women			0.745	0.966	0.859	0.021	0.825
Men			0.795	0.944	0.858	0.131	0.824
WHR							
Women				0.749	0.404	−0.104	0.242
Men				0.809	0.505	−0.075	0.316
WHtR							
Women					0.763	−0.236	0.772
Men					0.708	−0.201	0.723
Weight							
Women						0.271	0.908
Men						0.416	0.872
Height							
Women							0.119
Men							0.279

BMI indicates body mass index; WC, waist circumference; WHR, waist‐to‐hip ratio; WHtR, waist‐to‐height ratio.

**Figure 1:**
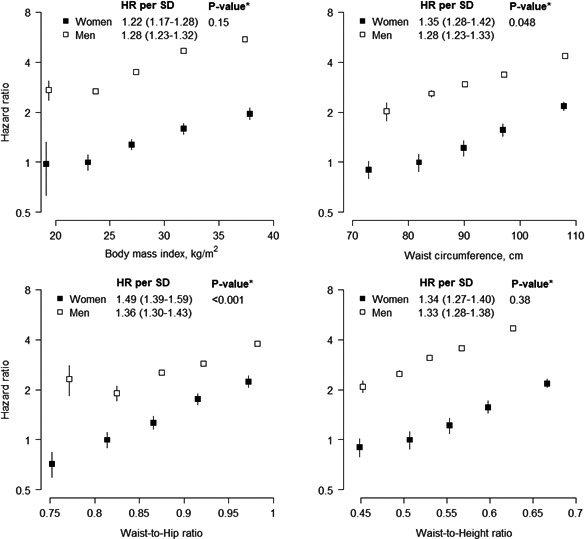
Adjusted hazard ratios (HRs) for incident myocardial infarction associated with body mass index, waist circumference, waist‐to‐hip ratio, and waist‐to‐height ratio. Analyses are adjusted for age, Townsend deprivation index, and smoking status. The first 2 years of follow‐up were excluded. HRs per fifth are plotted on a floating absolute scale, with the second fifth as the reference group. Vertical lines indicate the corresponding 95% confidence intervals. HRs for a 1‐SD higher value are shown for each sex, taking the standard deviation from the sex‐combined baseline data. *P‐value for interaction by sex for the continuous analysis.

During a mean follow‐up of 7.1 years, 5710 cases of MI (28% women) were recorded, including 1292 (25% women) events that occurred within 2 years of follow‐up. In both women and men there was an approximate log‐linear relationship between all measures of general and central adiposity and the risk of incident MI, notwithstanding a flattening of the association at lower levels of adiposity (Figure 1 and Table S2).

**Figure 2:**
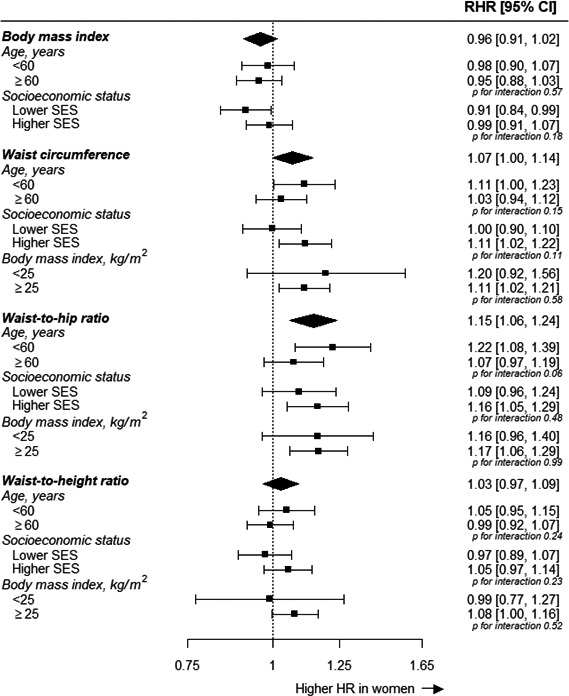
Ratio of women‐to‐men hazard ratios for incident MI associated with anthropometric measures, by age, socioeconomic status, and body mass index (where applicable). Analyses are adjusted for age, Townsend deprivation index, and smoking status, where appropriate. The first 2 years of follow‐up were excluded. CI indicates confidence interval; HR, hazard ratio; MI, myocardial infarction; RHR, ratio of hazard ratios; SES, socioeconomic status.

A 1‐SD higher BMI was associated with a HR for MI of 1.22 (1.17; 1.28) in women and 1.28 (1.23; 1.32) in men (*P* for interaction=0.15), with no evidence for differences in strengths or direction of the association by age or socioeconomic status (Table S3 and Figure 2). A 1‐SD higher waist circumference was more strongly associated with the risk of MI in women than in men, although the difference just reached statistical significance; the HR was 1.35 (1.28; 1.42) in women and 1.28 (1.23; 1.33) in men (*P* for interaction=0.048). The corresponding women‐to‐men ratio of HRs was 1.07 (1.00; 1.14). Although this sex difference was also observed among those aged <60 years at recruitment, those with a higher socioeconomic status, and in individuals with a BMI of 25 kg/m^2^ or above, but not among their counterparts, there was no statistical evidence for a 3‐way interaction between sex, waist circumference, and these subgroups (Figure 2). A 1‐SD higher waist‐to‐hip ratio was associated with a HR of MI of 1.49 (1.39; 1.59) in women and 1.36 (1.30; 1.43) in men (*P* for interaction=0.001), with a corresponding women‐to‐men ratio of HRs of 1.15 (1.06; 1.24). Although there was some variation in the magnitude of this sex difference across subgroups, these differences did not reach statistical significance. The association between a 1‐SD higher waist‐to‐height ratio and the risk of MI was similar between the sexes and did not vary materially by age, socioeconomic status, and BMI; the HR was 1.34 (1.27; 1.40) in women and 1.33 (1.28; 1.38) in men (*P* for interaction=0.38).

**Figure 3:**
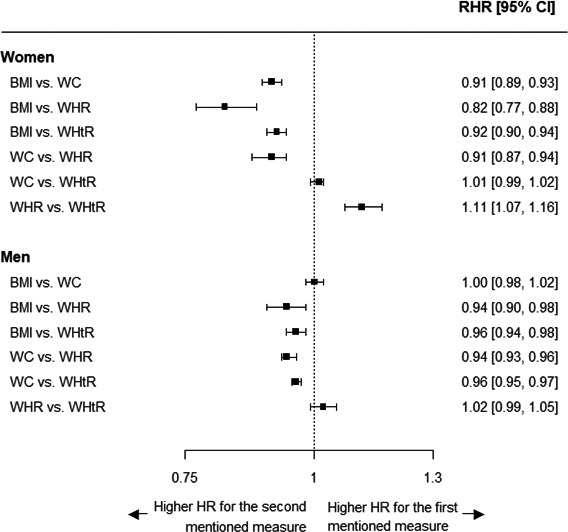
Ratio of women‐to‐men hazard ratios for incident MI per 1–standard deviation higher value for each comparison of anthropometric measures, by sex. Analyses are adjusted for age, Townsend deprivation index, and smoking status. The first 2 years of follow‐up were excluded. The standard deviation is taken from the sex‐combined baseline data. BMI indicates body mass index; CI, confidence interval; HR, hazard ratio; MI, myocardial infarction; RHR, ratio of hazard ratios; WC, waist circumference; WHR, waist‐to‐hip ratio; WHtR, waist‐to‐height ratio.

In women, higher values of central adiposity (ie, waist circumference, waist‐to‐hip ratio, and waist‐to‐height ratio) were associated with a 10% to 20% greater risk of MI than were higher values of BMI. Of these, waist‐to‐hip ratio was more strongly associated with MI than waist circumference and waist‐to‐height ratio (Figure 3). Differences in the strength of the association with MI across measures of adiposity were smaller in men. However, waist‐to‐hip ratio was also significantly more strongly associated with MI than BMI and waist circumference but not waist‐to‐height ratio.

## Discussion

This large prospective study among 480 000 women and men demonstrates that higher levels of measures of central and general adiposity are each associated with an increased risk of MI in later life. Waist circumference and waist‐to‐hip ratio, but not BMI and waist‐to‐height ratio, however, were more strongly associated with the risk of MI in women than in men. Moreover, measures of central adiposity, particularly waist‐to‐hip ratio, were more strongly associated with the risk of MI than BMI, especially among women. These findings were consistent across age and socioeconomic groups.

General and central adiposity have been well established as major modifiable risk factors for MI. However, studies have reported conflicting findings on the comparative association between different anthropometric measures and the risk of MI, with conflicting results for women and men. Analyses from the Emerging Risk Factors Collaboration among 222 000 individuals showed that BMI, waist circumference, and waist‐to‐hip ratio have similar strengths of association with coronary heart disease, with no evidence for sex differences in the relationships.^11^ Moreover, findings from the Physician's Health Study and the Women's Health Study have indicated that, although waist‐to‐height ratio was most strongly associated with the risk of cardiovascular disease, differences with BMI were small and were not considered as clinically meaningful in either sex.^12^ In contrast, the INTERHEART (Effect of Potentially Modifiable Risk Factors Associated with Myocardial Infarction) case‐control study including 12 500 cases of MI showed that, for both women and men, waist‐to‐hip ratio was strongly associated with the risk of MI, whereas the association of BMI with MI was considerably weaker and less consistent.^7^ Results from a study among 12 European cohorts and 2 Swedish studies each also suggested that measures of central obesity were more strongly associated with cardiovascular mortality than BMI, with some indication that the magnitude of the difference in the strength of the association across measures was greater in women than men.^9^, ^10^ Furthermore, the EPIC‐Norfolk (European Prospective Investigation of Cancer and Nutrition) study among 24 000 individuals showed that waist‐to‐hip ratio was more consistently associated with the risk of coronary heart disease than waist circumference or BMI in both women and men.^8^ The EPIC study including 360 000 individuals demonstrated that higher waist circumference (both sexes) and waist‐to‐hip ratio (women only) were associated with a higher risk of coronary death, independent of BMI.^6^ The findings from the present analyses complement and extend these previous studies in several important ways. First, using the same adiposity cut points in women and men in a single model facilitated direct comparisons of the risk of MI associated with adiposity measures in both sexes. Second, in addition to exclusively reporting sex‐specific effects, estimation of the magnitude of any potential sex differences revealed that waist‐to‐hip ratio and waist circumference, respectively, were 15% and 7% more strongly associated with the risk of MI in women than men. Third, direct quantitative comparisons among several measures of adiposity demonstrated that, for example, compared with BMI, waist‐to‐hip ratio was an 18% stronger predictor of MI in women and a 6% stronger predictor of MI in men. Waist‐to‐hip ratio may therefore be a more comprehensive indicator of the risk of MI associated with adiposity, especially in women but also in men.

This study suggests that the sex dimorphism in the quantity and distribution of adipose tissue not only results in differences in body shape between women and men but may also have differential implications for the future risk of MI. Body composition and fat distribution differ markedly between women and men, with a predominance of fat mass and subcutaneous fat in women and of lean mass and visceral fat in men.^13^, ^14^ These sex differences can, at least in part, be attributed to the influence of sex hormones on fat distribution. Although the major structural and functional differences between subcutaneous and visceral fat are well characterized, evidence for potential sex differences in the functionality of each type of adipose tissue is more limited.^13^, ^14^ However, genome‐wide association studies of markers of fat distribution, but not of BMI, have identified several sexually dimorphic variants, most of which conferred stronger effects in women than men.^19^, ^20^ The loci demonstrated to have a stronger effect in women than men included several genes known to be associated with lipid traits and insulin resistance,^20^ providing some biological basis for women's greater excess risk of MI associated with waist circumference and waist‐to‐hip ratio as seen in this study. Women develop diabetes mellitus at a higher level of BMI and are at a greater excess risk of stroke and coronary heart disease compared with men with diabetes mellitus.^21^, ^22^, ^23^, ^24^ Differences in the waist‐to‐hip ratio between individuals with and without diabetes mellitus, however, are broadly similar between women and men in the UK Biobank, providing further evidence that markers of fat distribution may be a better indicator of cardiometabolic risk among women than BMI.^25^

Our study has several strengths, including the prospective design, large sample size, and direct measurement of general and central adiposity on all participants. However, the UK Biobank is a largely white population, and further analyses are needed to determine the generalizability to other populations. Imaging‐derived measurements of body fat distribution and body composition will become available in the UK Biobank in due course^26^ and, combined with genetic data, will provide unique and detailed insights into the sex‐specific roles of different aspects of adiposity and the risk of MI and several other obesity‐related conditions. Further disentangling the sexual dimorphism in adiposity will yield insights into the biological mechanisms and could inform sex‐specific interventions to treat and halt the obesity epidemic worldwide.

In conclusion, although several measures of general and central adiposity each have profound deleterious effects on the risk of MI in both sexes, a higher waist‐to‐hip ratio and waist circumference conferred a greater excess risk of MI in women than men. Waist‐to‐hip ratio was more strongly associated with the risk of MI than BMI in both sexes, especially in women.

## Sources of Funding

Peters is supported by a UK Medical Research Council Skills Development Fellowship (MR/P014550/1).

## Disclosures

None.

## Supporting information


**Table S1.** Sex‐Specific Mean Differences (Standard Deviation) in Body Size Measures Between the Baseline Assessment and First Repeat Assessment
**Table S2.** Sex‐Specific Association of Body Mass Index, Waist Circumference, Waist‐to‐Hip Ratio, and Waist‐to‐Height Ratio With Incident MI
**Table S3.** Sex‐Specific Hazard Ratios, With 95% Confidence Intervals, for MI Associated With a 1‐SD Increase in Adiposity Measures by Age, Socioeconomic Status, and Body Mass Index
