# Supplementary material for: Sex Differences in the Association Between Measures of General and Central Adiposity and the Risk of Myocardial Infarction: Results From the UK Biobank
Source: J Am Heart Assoc. 2018 Feb 28;7(5):e008507. doi: 10.1161/JAHA.117.008507 (PMC5866342; doi:10.1161/JAHA.117.008507)
Supplement: Supplementary file 1 — Table S1. Sex‐Specific Mean Differences (Standard Deviation) in Body Size Measures Between the Baseline Assessment and First Repeat Assessment Table S2. Sex‐Specific Association of Body Mass Index, Waist Circumference, Waist‐to‐Hip Ratio, and Waist‐to‐Height Ratio With Incident MI Table S3. Sex‐Specific Hazard Ratios, With 95% Confidence Intervals, for MI Associated With a 1‐SD Increase in Adiposity Measures by Age, Socioeconomic Status, and Body Mass Index [file JAH3-7-e008507-s001.pdf]

SUPPLEMENTAL MATERIAL

Table S1. Sex-specific mean differences (standard deviation) in body size measures between the baseline assessment and first repeat assessment.

	Women	Men
**N**	10,374	9,903
**Body mass index, kg/m^2**	0.06 (1.94)	0.09 (1.52)
**Waist circumference, cm**	2.03 (7.08)	1.53 (6.60)
**Hip circumference, cm**	0.58 (5.80)	0.57 (4.74)
**Standing height, cm**	0.62 (1.10)	0.56 (1.11)
**Weight, kg**	0.39 (5.10)	0.25 (4.63)
**Waist-to-hip ratio**	0.02 (0.06)	0.01 (0.05)
**Waist-to-height ratio**	0.02 (0.04)	0.01 (0.04)

Table S2. Sex-specific association of body mass index, waist circumference, waist-to-hip ratio, and waist-to-height ratio with incident MI.

	Body mass index								
	<20	20-25	25-30	30-35	>35		Per sex-specific SD	Per sex-combined SD	P-value
Women	0.98 (0.63; 1.32)	1.00 (0.89; 1.11)	1.28 (1.19; 1.37)	1.59 (1.47; 1.71)	1.96 (1.81; 2.12)		1.24 (1.18; 1.31)	1.22 (1.17; 1.28)	0.15
Men	2.72 (2.36; 3.08)	2.68 (2.60; 2.77)	3.49 (3.44; 3.54)	4.68 (4.61; 4.75)	5.49 (5.37; 5.62)		1.24 (1.20; 1.28)	1.28 (1.23; 1.32)	
	**Waist circumference**								
	<78	78-86	86-93	93-101	>101				
Women	0.91 (0.79; 1.02)	1.00 (0.88; 1.12)	1.22 (1.09; 1.35)	1.57 (1.44; 1.70)	2.18 (2.05; 2.30)		1.32 (1.25; 1.39)	1.35 (1.28; 1.42)	0.048
Men	2.03 (1.77; 2.28)	2.59 (2.48; 2.71)	2.95 (2.87; 3.03)	3.36 (3.30; 3.43)	4.35 (4.29; 4.40)		1.23 (1.19; 1.27)	1.28 (1.23; 1.33)	
	**Waist-to-hip ratio**								
	<0.79	0.79-0.84	0.84-0.90	0.90-0.95	>0.95				
Women	0.72 (0.59; 0.84)	1.00 (0.90; 1.10)	1.27 (1.16; 1.38)	1.75 (1.62; 1.89)	2.24 (2.06; 2.42)		1.36 (1.30; 1.44)	1.49 (1.39; 1.59)	0.001
Men	2.31 (1.84; 2.79)	1.91 (1.72; 2.11)	2.54 (2.45; 2.63)	2.88 (2.82; 2.95)	3.78 (3.73; 3.83)		1.25 (1.21; 1.29)	1.36 (1.30; 1.43)	
	**Waist-to-height ratio**								
	<0.47	0.47-0.51	0.51-0.55	0.55-0.59	>0.59				
Women	0.90 (0.79; 1.02)	1.00 (0.88; 1.12)	1.22 (1.09; 1.35)	1.57 (1.44; 1.71)	2.18 (2.06; 2.31)		1.36 (1.29; 1.43)	1.34 (1.27; 1.40)	0.38
Men	2.09 (1.92; 2.25)	2.49 (2.39; 2.59)	3.12 (3.04; 3.20)	3.55 (3.49; 3.62)	4.67 (4.61; 4.73)		1.28 (1.24; 1.33)	1.33 (1.28; 1.38)	

Analyses are adjusted for age, Townsend deprivation index, and smoking status and excluded the first 2 years of follow-up

Table S3. Sex-specific hazard ratios, with 95% confidence intervals, for MI associated with a 1-SD increase in adiposity measures, by age, socioeconomic status, and body mass index.

	Women	Men	P-value for interaction by sex
	**Body mass index**		
Age, years			
<60	1.28 (1.19; 1.37)	1.30 (1.23; 1.37)	0.70
≥60	1.20 (1.13; 1.27)	1.26 (1.20; 1.32)	0.22
Socioeconomic status			
Lower SES	1.20 (1.12; 1.28)	1.30 (1.23; 1.37)	0.04
Higher SES	1.26 (1.18; 1.34)	1.27 (1.21; 1.33)	0.76
	**Waist circumference**		
Age, years			
<60	1.48 (1.35; 1.61)	1.33 (1.25; 1.41)	0.04
≥60	1.30 (1.22; 1.40)	1.28 (1.21; 1.35)	0.51
Socioeconomic status			
Lower SES	1.32 (1.22; 1.44)	1.34 (1.27; 1.43)	0.95
Higher SES	1.37 (1.28; 1.48)	1.25 (1.18; 1.31)	0.02
Body mass index, kg/m^2			
<25	1.37 (1.11; 1.68)	1.18 (1.00; 1.41)	0.19
≥25	1.36 (1.27; 1.47)	1.24 (1.18; 1.31)	0.02
	**Waist-to-hip ratio**		
Age, years			
<60	1.74 (1.56; 1.94)	1.45 (1.36; 1.54)	0.002
≥60	1.44 (1.33; 1.56)	1.37 (1.29; 1.46)	0.17
Socioeconomic status			
Lower SES	1.50 (1.36; 1.66)	1.45 (1.34; 1.56)	0.17
Higher SES	1.49 (1.36; 1.62)	1.32 (1.24; 1.40)	0.005
Body mass index, kg/m^2			
<25	1.42 (1.23; 1.65)	1.31 (1.16; 1.47)	0.11
≥25	1.46 (1.35; 1.59)	1.31 (1.23; 1.39)	0.002
	**Waist-to-height ratio**		
Age, years			
<60	1.46 (1.36; 1.58)	1.40 (1.32; 1.48)	0.33
≥60	1.30 (1.23; 1.38)	1.33 (1.27; 1.40)	0.81
Socioeconomic status			
Lower SES	1.33 (1.24; 1.42)	1.39 (1.31; 1.47)	0.56
Higher SES	1.35 (1.27; 1.44)	1.30 (1.24; 1.37)	0.21
Body mass index, kg/m^2			
<25	1.43 (1.19; 1.72)	1.59 (1.33; 1.89)	0.93
≥25	1.37 (1.29; 1.46)	1.30 (1.24; 1.36)	0.06

Analyses are adjusted for age, Townsend deprivation index, and smoking status and excluded the first 2 years of follow-up. SES, socioeconomic status. The UK median Townsend deprivation index of -0.56 was used to categorise individuals as ‘Lower (> -0.56)’ or ‘Higher (≤ -0.56)’ SES.
